# Factors associated with a high level of unmet needs and their prevalence in the breast cancer survivors 1–5 years after post local treatment and (neo)adjuvant chemotherapy during the COVID-19: A cross-sectional study

**DOI:** 10.3389/fpsyg.2022.969918

**Published:** 2022-10-03

**Authors:** Špela Miroševič, Judith Prins, Simona Borštnar, Nikola Besić, Vesna Homar, Polona Selič-Zupančič, Andreja Cirila Škufca Smrdel, Zalika Klemenc-Ketiš

**Affiliations:** ^1^Department of Family Medicine, Faculty of Medicine, University of Ljubljana, Ljubljana, Slovenia; ^2^Department of Medical Psychology, Radboud University Medical Centre, Nijmegen, Netherlands; ^3^Department of Medical Oncology, Institute of Oncology, Ljubljana, Slovenia; ^4^Department of Surgical Oncology, Institute of Oncology, Ljubljana, Slovenia; ^5^Department of Psychology, Faculty of Medicine, University of Maribor, Maribor, Slovenia; ^6^Community Health Centre Ljubljana, Primary Healthcare Research and Development Institute, Ljubljana, Slovenia; ^7^Department of Psycho-Oncology, Institute of Oncology Ljubljana, Ljubljana, Slovenia; ^8^Department of Family Medicine, Medical Faculty, University of Maribor, Maribor, Slovenia

**Keywords:** breast cancer, cancer survivors (MeSH term), needs assessment [MeSH], fear of cancer recurrence, quality of life, psycho-oncology

## Abstract

**Objective:**

To assess the prevalence of unmet needs in post-treatment breast cancer survivors and identify sociodemographic, clinical, and psychosocial variables associated with reported unmet needs during the COVID-19 pandemic.

**Materials and methods:**

In this cross-sectional study, 430 post-treatment breast cancer survivors, ranging between 1 and 5 years after the procedure, completed the Cancer Survivors’ Unmet Needs (CaSUN) questionnaire from September 2021 and January 2022. The multivariate logistic analysis identified factors associated with at least one reported unmet need in the total CaSUN scale and specific domains.

**Results:**

A total of 67% of survivors reported at least one unmet need. The most frequently reported unmet needs were the lack of accessible hospital parking (43%) and recurrence concerns (39.5%). The majority of reported unmet needs relate to comprehensive care (44%), followed by the psychological and emotional support domain (35.3%). Younger age (OR = 0.95, 95% CI = 0.92–0.99; *p* < 0.001), three or more comorbidities (OR = 0.27, 95% CI = 0.11–0.71, *p* < 0.01), a lower quality of life (OR = 0.06, 95% CI = 0.01–0.47, *p* < 0.01) and low resilience (OR = 0.95, 95% CI = 0.93–0.99) were associated with a high level of unmet needs in the multivariate regression model. Results are presented for factors associated with a high level of unmet needs for comprehensive cancer care and psychological and emotional support domain.

**Conclusion:**

A high prevalence found in our study could be attributed to the COVID-19 pandemic, where patients may have missed adequate follow-up care, although comparing to studies done in non-pandemic time is difficult. Family physicians should be more attentive toward younger cancer survivors and those with more comorbidities as both characteristics can be easily recognized in the family practice.

## Introduction

The COVID-19 pandemic has changed the lives of cancer survivors. Cancer survivors are a vulnerable group that is more at risk of having a severe disease course (Kuderer et al., 2020). Due to this vulnerability, many cancer treatments and appointments have been delayed or modified. In addition, social distancing profoundly impacted the quality of life of some cancer survivors, worsened their quality of life (Schandl et al., 2021), and heightened the levels of psychological distress and fear of cancer recurrence (Ayubi et al., 2021; Zomerdijk et al., 2021; Kim and Kim, 2022).

In European countries, the 5-year survival rate for women with breast cancer (BC) has improved significantly; nevertheless, BC survivors face many stressors daily, including physical discomfort (e.g., post-treatment chronic pain, fatigue, menopausal symptoms), psychosocial (e.g., fear of cancer recurrence (FCR), accepting body changes), and practical challenges (e.g., returning to work, taking care of family) associated with the diagnosis and the treatment (Gulluoglu et al., 2006; Stanton, 2012; Mirosevic et al., 2019b; Shakeel et al., 2020). Measuring unmet needs differentiates from measuring patients’ symptoms as it focuses on identifying the unresolved concerns that a patient may experience and detect if and in what severity they require further assistance (Sanson-Fisher et al., 2000). Unmet needs are essential from a scientific and clinical view, as they can inform who are those individuals with more unmet needs to improve cancer management and enable the development of tailored interventions.

According to the systematic review published in 2019, breast cancer (BC) survivors, compared to other types of cancer, experience a high prevalence of unmet needs (Mirosevic et al., 2019a). A significant level of supportive needs was found even after 10 years (Burg et al., 2015). Previous studies done before the pandemic report that the prevalence of unmet needs of BC survivors ranged from 49% to 88% (Hodgkinson et al., 2007b; Park and Hwang, 2012; Cheng et al., 2014, 2016; So et al., 2014; Brennan et al., 2016; Fang et al., 2018) and the most commonly endorsed unmet need was FCR, followed by information needs (Mirosevic et al., 2019a). In the multivariate regression models, younger age (von Heymann-Horan et al., 2013; Brennan et al., 2016), shorter time since the end of the treatment (Cheng et al., 2016), more symptoms of anxiety (Hodgkinson et al., 2007b; von Heymann-Horan et al., 2013), a higher number of physical symptoms (Cheng et al., 2014; Fang et al., 2018), lower quality of life (Park and Hwang, 2012), and a higher FCR score (Fang et al., 2018) were associated with at least one reported unmet need.

While the variables mentioned above seem to be well established, a recent systematic review on the topic (Mirosevic et al., 2019a) was not conclusive about how time post-treatment, stage of the disease at diagnosis and depression are associated with high levels of unmet needs. Additionally, a few factors are potentially related to unmet needs, such as marital status (von Heymann-Horan et al., 2013), education (von Heymann-Horan et al., 2013), employment status (Chae et al., 2019), smoking status (Rowlands et al., 2015), cancer stage (Chou et al., 2020), hormonal therapy (Fang et al., 2018) and treatment type (Lo-Fo-Wong et al., 2020), worth exploring further.

To our knowledge, few studies on breast cancer survivors explore how resilience and social support impact the severity of unmet needs. Resilience has become an important construct in adjusting to cancer and the pandemic recently. Cancer survivors may have been better prepared for the existential distress caused by the pandemic due to their experience with the cancer diagnosis. Resilience was found to be negatively associated with unmet needs (Dubey et al., 2015; Mirosevic et al., 2022a). Regarding social support, breast cancer patients, compared to individuals with other cancers, have better opportunities to receive more information support due to their vast active patient advocacy network. However, those with low social support are less likely to get the help and information they need (Arora et al., 2007).

The current study was exploratory, and the above variables were selected based on the literature reports on variables associated with high unmet needs. This study aimed to explore the BC survivors’ unmet needs during the pandemic and identify differences in results from previous studies that were done before the pandemic. Specifically, to (a) determine the prevalence of at least one reported unmet need in the total Cancer Survivors’ Unmet Needs (CaSUN) Scale and specific domains, and (b) assess the demographic, clinical, psychological, and behavioral factors associated with at least one reported unmet need.

## Materials and methods

This cross-sectional study was approved by the National Medical Ethics Committee (no. 0120-25/2019/6) and the Ethics Committee of the Institute of Oncology Ljubljana (EK-OI-16092021). Furthermore, the study protocol was in compliance with the 1964 Declaration of Helsinki and its later amendments for recommendations guiding physicians in biomedical research involving human subjects.

Female BC survivors who met the inclusion criteria and attended a follow-up appointment at the Institute of Oncology Ljubljana between September 2021 and January 2022 were invited to participate in the study. Inclusion criteria were (a) diagnosed with BC after the age of 18, (b) diagnosed and treated for primary BC, (c) 1–5 years post-primary treatment with surgery +/− radiotherapy +/− chemotherapy, (d) without signs of recurrence, (e) speak and write fluent Slovene, and (f) no cognitive disorders that could hinder the results of completing the questionnaire. Participants were informed about the nature of the study and signed the informed consent. The main researcher of the study was present with the participants most of the time; thus, if any misunderstanding occurred while completing the questionnaire, the researcher could explain the question and resolve any misunderstandings.

### Study instruments

#### Cancer survivors’ unmet needs

Supportive care needs were assessed with a widely used measure, Cancer Survivors’ Unmet Needs (CaSUN) (Hodgkinson et al., 2007a). Each question asks about the presence of a specific need and its strength in the past months. Prior to this study, a translation and validation of the scale was done in a separate study (Mirosevic et al., 2022b). The exploratory analysis of the Slovenian version of the CaSUN revealed that 27 items fall within one of the five domains: existential survivorship (7 items), comprehensive cancer care (7 items), psychological and emotional support (7 items), relationship (3 items), and information (3 items). A total of 7 items were not included in the analysis and the domains, however they were included in the final version of the measure in the total needs score (see validation study for more information) (Mirosevic et al., 2022a). The Slovenian translated and localised version is a valid and reliable measure with 34 items (Mirosevic et al., 2022b). Cronbach’s alpha calculated for the present sample was found to be adequate to excellent in all five domains (existential survivorship, *α* = 0.83; comprehensive cancer care, *α* = 0.87; psychological and emotional support, *α* = 0.86; relationship, *α* = 0.71; information, *α* = 0.73) and in the total 34-item version (*α* = 0.94). For this study, unmet needs were classified as no need (no need, met need or weak need) and unmet need (moderate or strong need) for the total 34-item score and for each of the above-written domains (see Methods for reasons).

#### Sociodemographic and clinical variables

A study specific questionnaire included assessing information about age, marital status, education, employment status, place of residence, smoking status, cancer stage, type of primary treatment (surgery only, surgery and adjuvant chemotherapy, surgery and radiotherapy or surgery and chemotherapy and radiotherapy), presence of hormonal treatment and time (in months) since the end of primary treatment.

#### Presence of comorbidities

The self-report Comorbidity Questionnaire (SCQ-19) consists of 16 common and three optional comorbidities (Sangha et al., 2003). Items are scored as a binary response to the following questions: Do you have a problem?, Are you receiving treatment for it?, and Does it limit your activities?. The SCQ-19 is a validated and reliable measure that has been commonly used in the research of cancer patients (Robinski et al., 2016; Rohrl et al., 2019). For this study, the following categorization was used: no comorbidities, 1–2 comorbidities, or more than 3 comorbidities.

#### Symptoms of anxiety and depression

The Hospital Anxiety and Depression Scale (HADS) is a 14-item self-reported combined measure assessing symptoms of anxiety (HADS-A) and depression (HADS-D). Each subscale is scored on a 4-point Likert scale, ranging from 0 to 21. The recommended screening cut-off score of 11 indicates clinical cases of anxiety/depression (Zigmond and Snaith, 1983). The Slovenian version of the HADS validated in female cancer patients shows good psychometric properties (Miklavcic et al., 2008). The Cronbach’s alpha for the total distress score, anxiety and depression subscale show excellent-good values at 0.92, 0.86, and 0.87, respectively.

#### Quality of life

The EuroQol Five-Dimension Questionnaire (EQ-5D) is an instrument that was developed in Europe and is widely used for measuring the generic quality of life. It includes five dimensions, including mobility, self-care, usual activities, pain/discomfort, and anxiety/depression, each with five different severities (no/slight/moderate/severe/extreme problems or unable to perform the activity). In addition, participants described their current health status, presented in a visual analogue scale (EQ-VAS). Score 0 represents the worst, and score 100 the best health status they can imagine (EuroQol, Group, 1990). The Slovenian version of the EQ-5D was adequately validated on a Slovenian sample of elderly patients with diabetes mellitus type 2 (Turk et al., 2014). The Cronbach’s coefficient value calculated for this study was adequate (*α* = 0.72). For this study, utility values were calculated according to the Slovenian population norm (EQ5DL-crosswalk-SL; data not published yet).

#### Fear of cancer recurrence

The Fear of Cancer Recurrence Inventory (FCRI) is a self-reported 42-item scale that measures patients’ level of FCR. It comprises seven subscales, including severity, triggers, psychological distress, coping strategies, insight, reassurance behavior, and overall functioning. Those are rated on a 5-item Likert scale. The Slovenian version of the FCR has been forward-backward translated and validated prior to this study (results not published yet). The Cronbach’s alpha of the FCRI total score (*α* = 0.95) and the severity subscale (*α* = 0.86) for the present sample was calculated. In this study only the severity subscale is used as it is the most commonly used scale and reported to be the most representative of the FCR levels (Costa et al., 2016; Liu et al., 2018).

#### Resilience

The 14-item Resilience Scale (FCR) measures a person’s ability to successfully recover from stressful events (Masten et al., 1990). The RS-14 is a widely used scale that has been translated into several languages and validated on various samples, including cancer survivors (Tian and Hong, 2013; Mirosevic et al., 2018). Items are rated on a 7-point Likert scale and each item is graded from 1 (strongly disagree) to 7 (strongly agree). Items are summed together and represent the RS-14 total score. The Slovenian version of the RS-14 was adequately validated prior to the study (Mirosevic et al., 2022a). A Cronbach’s alpha of the present study was found to be excellent (*α* = 0.92).

#### Social support

The Multidimensional Scale of Perceived Social Support (MSPSS) is a 12-item self-administered scale that measures how a person perceives social support. Social support is measured by three subscales: Significant Others (SO), Family (FA), and Friends (FR). The participants are asked to indicate their level of agreement with each item (1 – very strongly disagree, 7 – very strongly agree). The scores of each subscale (score range 1–4) are totaled; higher scores indicate better perceived social support. The scale was translated, back-translated and validated in a sample of Slovenian students (Ivić, 2017). Cronbach’s alpha calculated for this sample was 0.95, 0.97, 0.96, and 0.96 for the sub-scale SO, FA, FR and the total MSPSS score, respectively.

### Statistical analysis

#### Sample size calculation

This study was planned with the information that the prevalence of at least one reported unmet need in the sample of BC survivors as reported in the literature ranges from 49% to 88% (Mirosevic et al., 2019a). In the present sample, 67% of the participants reported having at least one unmet need. The sample size of 430 cancer survivors includes 288 participants with at least one reported unmet need. This allows the inclusion of at least 28 variables if we consider the rule of thumb for logistic regression, which states that there must be 10 events on one variable (Peduzzi et al., 1996).

#### Missing values

Missing values for the CaSUN were handled according to the guidelines (Hodgkinson et al., 2007a). The missing values on each CaSUN items were marked with ‘no need/does not apply to me’ and counted as zero in the total unmet needs score and in each specific domain; but if all the values were missing for any of the domains, the whole set of items were marked as missing, and the case was excluded (Smith et al., 2013; Willems et al., 2016). Then, the FCRI was checked to ascertain if the same response pattern was used for all the items of the FCRI, especially item 13 that is reversed, as this indicates an automated response (Monette et al., 2013; van Helmondt et al., 2021). Then, for all the other variables, we ran the pattern analyze and handled missing values using multiple imputation analysis.

#### Descriptive statistics and multiple logistic analysis

The prevalence of unmet needs was assessed using different types of descriptive statistics: (a) the percentage of cancer survivors with at least one unmet need (moderate or strong); (b) the percentage of at least one unmet need (moderate or strong) according to each specific domain; (c) the 10 most frequently reported unmet and met needs and the mean value of their strength; and (d) the mean value of the total number of unmet needs and for each domain. We categorized cancer survivors into a group of ‘no unmet needs’, including no needs, met needs, and weak needs (which we named “low needs”); and a group of ‘at least one unmet need’, including moderate and strong unmet needs (which we named “high needs”). The reason for this decision was to distinguish between those who are only slightly dissatisfied from those whose needs are a bigger concern, which follows previous studies (Harrison et al., 2011; Molassiotis et al., 2017). Age was found to be a strong predictor of unmet needs (Mirosevic et al., 2019a), thus, we used the age-adjusted univariate analysis to explore the association between potential predictors and ‘at least one unmet need’. Variables statistically significant at the 0.2 level in the age-adjusted univariate analysis were further analyzed in the multivariate hierarchical binary logistic regression. The final model included variables that were statistically significant at the 0.05 level. In addition, when the prevalence of specific domains was above the threshold (i.e., reported by at least 30% of the sample), we used the same analysis to calculate which variables are associated with each of the five domains of the CaSUN scale.

## Results

**Figure 1:**
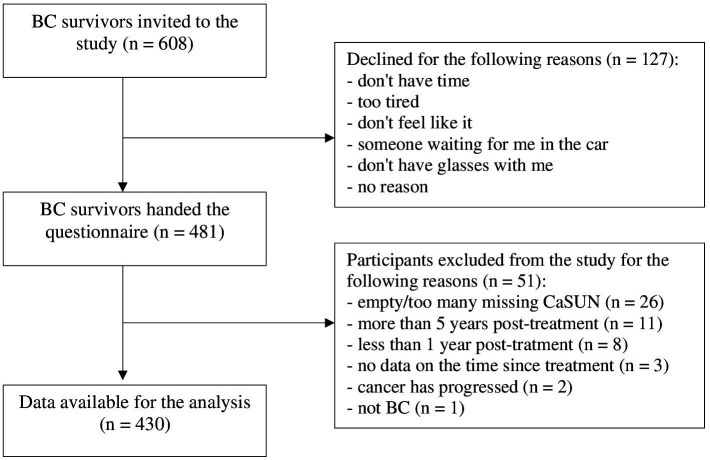
Study process.

Figure 1 shows the study process of recruitment and retention. Of the 608 invited participants, 127 declined the invitation, which indicates a good participation rate (79.0%). Participants’ reasons were noted (see Figure 1 for reasons). A pattern analysis for handling our missing data showed that the values are randomly arranged; there is no pattern. Twenty-three percent of the variables have missing values, 44% of the cases have at least one missing variable. Altogether, there are 1.78% of the missing values in our sample. After excluding the questionnaires of participants who did not fit the inclusion criteria or had too many missing values on the CaSUN, 430 BC survivors’ sets of data were considered for analysis.

### Patients’ characteristics

The mean age of the participants was 55.5 (standard deviation (*SD*) = 12.4), the majority of them were married (62.0%), diagnosed with stage II cancer (58.6%) and 29.9 months post primary treatment (*SD* = 18.2). The rest of the sociodemographic, disease- and treatment-related, and psychological variables are shown in Table 1.

**Table 1:** Sociodemographic, disease and treatment-related, and psychological variables (*N* = 430).

Characteristics	Sample of BC survivors
Age (mean ± *SD*), range: 18–90	55.5 (12.4)
Marital status (*n*, %)	
Married	266 (61.9)
Partnered	66 (15.3)
Single, divorced	60 (14.0)
Widowed	38 (8.8)
Education (*n*, *%*)	
Primary Education	28 (6.5)
Secondary Education	194 (45.1)
University, PhD	208 (48.4)
Employment (*n*, *%*)	
Full-time employed	171 (39.8)
Half-time employed	86 (20.0)
Retired	146 (34.0)
Disabled retired	14 (3.3)
Unemployed	13 (3.0)
Place of residence (*n*, *%*)	
Urban	161 (37.4)
Suburban	155 (36.0)
Rural	114 (26.5)
Smoking status (*n*, *%*)	
Yes	41 (9.5)
No	313 (72.8)
No, but smoked in the past	76 (17.7)
Cancer stage (*n*, *%*)	
0–I	90 (20.9)
II	252 (58.6)
III	88 (20.5)
Primary treatment, besides surgery (*n*, *%*)	
Chemotherapy (C)	48 (11.6)
Radiotherapy (R)	169 (39.3)
C and R	163 (37.9)
None	50 (11.6)
Time since treatment (mean ± SD), range: 8–66 months	29.9 (18.2)
Hormonal therapy (*n*, *%*)	
Yes	274 (63.7)
No	156 (36.3)
Comorbidities (SCQ-19; *n, %)*	
None	172 (40.0)
1–2 comorbidities	198 (46.0)
≥ 3 comorbidities	60 (14.0)
Psychological distress (HADS; mean ± *SD*)	
Anxiety	6.1 (3.6)
Depression	5.9 (3.9)
Fear of Cancer Recurrence (FCRI; mean ± *SD*)	14.0 (7.0)
Quality of Life (EQ-5D– index; mean ± *SD*)	0.74 (0.17)
Resilience (RS-14; mean ± *SD*)	80.8 (12.6)
Social support (MPSS–total; mean ± *SD*), missing, n = 8	68.8 (15.3)

BC, breast cancer; SD, standard deviation; HADS, The Hospital Anxiety and Depression Scale; SCQ-19, The self-administered comorbidity questionnaire; FCRI, Fear of Cancer Recurrence Inventory, EQ-5D, The EuroQol Five-Dimension Questionnaire; RS-14, The 14-item resilience scale; MPSS, The Multidimensional Scale of Perceived Social Support.

### Prevalence of unmet and met needs

**Figure 2:**
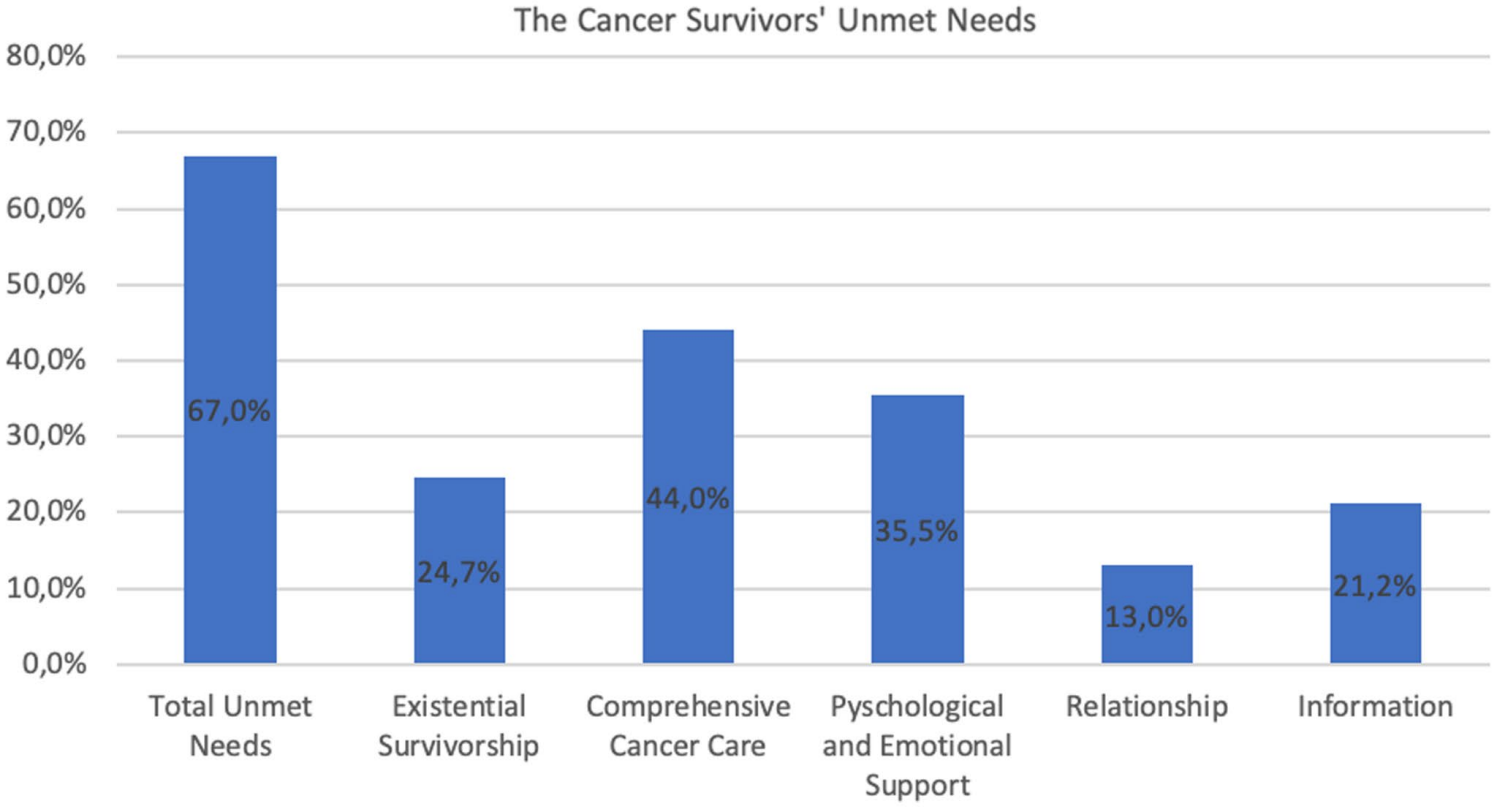
The prevalence of the total unmet needs and the unmet needs in each specific domain.

The prevalence of high unmet needs was 67% for the total CaSUN scale. The mean number of the total unmet needs was 4.3 (*SD* = 6.0, range = 0–33). The prevalence of high unmet needs for the existential survivorship, comprehensive cancer care, psychological and emotional support, relationship and information domain was 24.7%, 44%, 35.3%, 13%, and 21.2%, respectively (see Figure 2). The mean number of unmet needs for each specific domain were: existential survivorship 0.61 (*SD* = 1.3, range = 0–7), comprehensive cancer care 1.2 (*SD* = 1.8, range = 0–7), psychological and emotional support 1.04 (*SD* = 1.9, range = 0–7), relationship 0.2 (*SD* = 0.6, range = 0–3) and information 0.4 (*SD* = 0.8, range = 0–3). Table 2 shows the 10 most frequently reported unmet needs and their strengths.

**Table 2:** Ten most frequently reported unmet needs and its strength.

CaSUN item	Domain	Frequency, *n* (*%*)	Strength (*M* ± *SD*)
Accessible hospital parking	CC	185 (43.0)	2.30 (0.80)
Recurrence concerns	PES	170 (39.5)	1.80 (0.79)
Reduce stress	PES	154 (35.8)	1.71 (0.71)
Local health care services	CC	144 (33.5)	1.98 (0.82)
Manage side effects	CC	143 (33.3)	1.77 (0.70)
Complementary therapy	CC	142 (33.0)	2.04 (0.82)
Emotional support	PES	135 (31.4)	1.74 (0.80)
Doctors talk to each other	CC	129 (30.0)	1.87 (0.83)
Manage health with teams	CC	122 (28.4)	1.79 (0.75)
Ongoing case manager	CC	121 (28.1)	1.85 (0.88)

CaSUN, cancer survivors’ unmet need; CC, comprehensive care; PES, psychological and emotional support; M, mean; SD, standard deviation.

Almost all cancer survivors reported at least one met need (97.2%). The mean number of met needs was 14.3 (SD = 9.6). The most common met needs related to comprehensive cancer care (i.e., best medical care, managing health with teams, doctors talking to each other) and information (i.e., understandable information, up to date information). See Table 4 for detailed information.

### Factors associated with at least one reported unmet need

Results of the age-adjusted univariate analysis are presented in Supplement 1. When controlling for age, patients with high unmet needs in the total CaSUN scale are more likely to be single and divorced as compared to the married, have lower levels of education, are half-time employed, were more likely to have three or more comorbidities, have symptoms of anxiety and depression, experience a lower quality of life, have lower resilience and lower social support, and have higher levels of FCR. Only two domains—comprehensive care and psychological and emotional support domain, had a prevalence higher than 30%; thus, both were further explored in the univariate age-adjusted analysis (see Table 3).

**Table 3:** Breast cancer survivors’ 10 most common met needs.

CaSUN item	Domain	Frequency, *n* (*%*)
Best medical care	CC	271 (63%)
Manage health with teams	CC	269 (62.6%)
Understandable information	IN	264 (61.4%)
Doctors talk to each other	CC	244 (56.7%)
Up to date information	IN	232 (54%)
Local health care services	CC	228 (53%)
Talk to others	ES	237 (55.1%)
Move on with my life	PES	226 (52.6%)
Decisions about my life	PES	204 (47.4%)
Changes to belief	PES	199 (46.3%)

CaSUN, cancer survivors’ unmet need; CC, comprehensive care; PES, psychological and emotional support; M, mean; SD, standard deviation.

Multicollinearity was checked prior to conducting the multivariate analysis. The VIF was below 10, indicating no multicollinearity problems. The final regression models are shown in Table 4. Patients with a high level of unmet needs in the total CaSUN scale were more likely to be younger, have three or more comorbidities as compared to none, have a lower quality of life and lower resilience (*χ*^2^ = 125.749, df = 23; *p* < 0.001). Survivors with a high level of unmet needs in comprehensive cancer care were more likely to be younger, have less time passed since the end of the treatment, have three or more comorbidities as compared to the none, and have a lower quality of life (*χ*^2^ = 112.111, df = 19; *p* < 0.001). Patients with high unmet needs in the psychological and emotional support domain were more likely to be younger, experience more symptoms of anxiety, have a higher level of FCR, and have less perceived social support (*χ*^2^ = 186.256, df = 18; *p* < 0.001).

**Table 4:** Final logistic regression models predicting total unmet needs and unmet needs in the comprehensive care and psychological and emotional support domain.

Variable	Total unmet needs	Comprehensive cancer care	Psychological and emotional Support
	OR (95% CI)	OR (95% CI)	OR (95% CI)
Age	0.96 (0.93–0.99)*	0.96 (0.93–0.99)*	0.93 (0.90–0.97)**
Marital status (ref. married)			
Partnered	1.17 (0.56–2.44)		
Single, divorced^a^	1.95 (0.88–4.31)		
Widowed^a^	1.30 (0.51–3.33)		
Education (ref. Primary)			
Secondary^a^	0.53 (1.17–1.60)		0.90 (0.29–2.87)
University, PhD^a^	0.40 (0.12–1.30)		0.94 (0.28–3.13)
Employment status (ref. full-time)			
Half-time^a^	1.43 (0.68–2.99)		
Unemployed^a^			0.18 (0.03–1.13)
Disabled retired^a^	0.80 (0.14–4.60)	1.80 (0.42–7.74)	1.26 (0.28–5.73)
Place of residence (ref. urban)			
Sub-urban^a^			1.73 (0.94–3.19)
Rural^a^			1.42 (0.73–2.76)
Smoking status (ref. Never smoked)			
Currently smoking^a^		0.57 (0.25–1.30)	
Time since treatment	0.99 (0.97–1.00)	0.98 (0.97–0.99)*	
Cancer stage (ref. 0-I)			
III^a^	2.01 (0.88–4.59)		
Treatment type (ref. None)			
Chemotherapy (C)^a^		1.97 (0.78–5.02)	1.89 (0.65–5.54)
C + R^a^		1.27 (0.61–2.65)	0.95 (0.40–2.24)
Hormonal therapy (yes, no)		1.35 (0.84–2.17)	
SCQ-19, no. of comorbidities (ref. ≥ 3)			
None^a^	0.35 (0.14–0.84)*	0.32 (0.14–0.73)**	0.59 (0.23–1.53)
1–2^a^	0.54 (0.24–1.19)	0.67 (0.33–1.36)	0.97 (0.41–2.28)
EQ-5D - index, quality of life	0.10 (0.02–0.67)*	0.18 (0.03–0.99)*	0.25 (0.03–2.18)
HADS, anxiety	1.03 (0.92–1.15)	0.99 (0.89–1.10)	1.12 (1.03–1.30)*
HADS, depression	1.04 (0.93–1.20)	1.07 (0.97–1.18)	1.03 (0.92–1.15)
FCRI, fear of cancer recurrence	1.02 (0.97–1.07)	1.02 (0.98–1.06)	1.07 (1.02–1.13)**
RS-14, resilience	0.96 (0.93–0.99)*	0.98 (0.96–1.00)	0.98 (0.95–1.00)
MPSS-total, social support	0.98 (0.96–1.00)	1.00 (0.98–1.02)	0.97 (0.95–0.99)**

OR, odds ratio; CI, confidence interval.

a Predictor is a dumm y variable.

*p < 0.05, **p < 0.01.

## Discussion

This study provides a comprehensive examination of unmet needs and factors associated with high levels of unmet needs in BC survivors 1–5 years after primary treatment. In this cross-sectional study, we found a high prevalence of unmet needs (67%). While comparison with other studies is difficult, the high prevalence rate observed in this study might pertain to the missed follow-up care during the COVID-19 pandemic. Unmet needs were reported across all domains, including comprehensive cancer care (44%), psychological and emotional support (35.3%), existential survivorship (24.7%), information (21.2), and relationship (13%). The most common unmet needs concerned practical unmet needs (e.g., accessible hospital parking and accessing local health care services), needs related to psychological and emotional issues (e.g., fear of cancer recurrence, reducing stress and emotional support), and needs concerning management of healthcare and complications (e.g., managing side effects, complementary therapy, ongoing case manager). Younger age, a higher number of comorbidities, a lower quality of life and lower resilience were significant predictors of having high levels of total unmet needs.

A high prevalence found in our sample could be attributed to the fact that since the data was collected during the fourth wave of the pandemic, these patients may have missed adequate follow-up care in the first, second and third wave. Previous studies done in BC survivors with less than 5 years post-treatment reported that the prevalence of unmet needs ranges from 49% to 88% (Cheng et al., 2014, 2016; So et al., 2014; Brennan et al., 2016; Ellegaard et al., 2017; Fang et al., 2018). While our study prevalence falls within this range, the comparison between other studies done in non-pandemic time is difficult due to the large variations in the time since the diagnosis/treatment (range = 1–15 years), varying health-care systems, and the fact that the prevalence in reported studies was not uniformly assessed. Future data analyses using this sample might explore if, with time, unmet needs in breast cancer survivors will decrease once the hospital activity is continuously stable.

Compared to other types of cancer, the high prevalence found in ours and other BC studies reflects the high needs that might be more uniquely related to the specific population explored here. One reason might be the relatively low mean age (51–56 years) of BC survivors in reported studies, including ours, as compared to the mean age (60–72 years) of patients with mixed or other types of cancers including head and neck cancer, haematological cancer, prostate cancer, and colorectal cancer. It was reported that testicular cancer patients (mean age 35–38 years) have the highest prevalence of unmet needs among all cancers, i.e., in 62–68% of cases (Bender et al., 2012; Smith et al., 2013). This is not a surprise, as age has been found to be one of the strongest predictors of having unmet needs in the multivariate regression models in other studies (Harrison et al., 2011; Smith et al., 2013; Rowlands et al., 2015; Santin et al., 2015; Brennan et al., 2016; Molassiotis et al., 2017; Mirosevic et al., 2019a) and as well in ours. Younger BC survivors are faced with many challenges, including fertility problems, returning to work while still raising children, and a number of treatment-related adverse events, including weight gain, chronic fatigue, osteoporosis, cognitive dysfunctions, and hot flushes.

In addition to younger age, three or more comorbidities, poor quality of life, and lower resilience were associated with higher levels of unmet needs. While more symptoms (Cheng et al., 2014), other chronic illness (Smith et al., 2013), two or more comorbidities (Rowlands et al., 2015), number of symptoms (Fang et al., 2018) and poor quality of life (Santin et al., 2015; Cheng et al., 2016; Willems et al., 2016; Molassiotis et al., 2017) are known predictors in multivariate regression models, less evidence is known for the concept of resilience. Resilience is a relatively new concept in healthcare with the first measures having been developed in 1990 (Ahn, 1991). In cancer care, resilience has received even less attention (Bowen et al., 2003), albeit results from studies of resilience in cancer survivors show that more resilient survivors adapt better to disease and its consequences (Rowland and Baker, 2005). A recent systematic review and meta-analysis explored the benefits of interventions to promote resilience and found beneficial effects, especially in those interventions that lasted more than 12 sessions and included patients with an acute disease (Ludolph et al., 2019). Thus, based on already existing and evidence-based data from the literature, interventions that promote resilience should be considered to increase its benefit.

Furthermore, survivors with a high level of unmet needs in the psychological and emotional support domain were more likely to have more symptoms of anxiety, lower perceived social support and higher levels of FCR. To our knowledge, this is the first study conducted on BC survivors which reports that perceived social support is an important predictor of a high level of unmet needs in this specific domain; previous reports were completed on patients with other types of cancers, including endometrial and mixed cancers (Faghani et al., 2015; Rowlands et al., 2015). Symptoms of anxiety (Hodgkinson et al., 2007b; Harrison et al., 2011; Smith et al., 2013; Oberoi et al., 2017) and FCR (Armes et al., 2009; Urbaniec et al., 2011; Fang et al., 2018) were already established variables associated with a high level of unmet needs.

The most frequently reported unmet need was the lack of accessible hospital parking, which is something hospitals should be taken care of since cancer patients usually have a significant physical disability. Fear of cancer recurrence was the second highest endorsed unmet need, indicating an important need that demands special attention. It is important to mention some of the unmet needs that may have arisen due to the COVID-19 pandemic. Specifically, one of the strongest unmet needs during the time of the pandemic was limited personal access to local healthcare services. While an oncologist takes good care of patients’ physical concerns (e.g., management of treatment side effects, breast examination, disease control), survivors tend to seek emotional support more from their general practitioners (GP) (Hoekstra et al., 2014; Tompkins et al., 2016), which was practically infeasible during the pandemic. As the pandemic is currently on the decrease and GPs are more approachable, future data analysis might examine if this need decreases in time.

There were also several variables found to be non-significant in the multivariate models. Most of them were significant at the 0.05 level in the age-adjusted univariate model but no longer in the multivariate one, which may indicate that they are so-called proxy variables. In future research, it may be better to focus on those variables that were significantly associated with the outcome in the multivariate models because, in this way, we select the characteristics that have a more direct effect on the outcome.

### Study limitations

This study attempted to overcome the limitations found in previous studies (Mirosevic et al., 2019a). This includes exploring factors associated with not only total unmet needs, but also the needs in specific domains, providing information about cancer stage, the time since the end of treatment, and including a relatively large (*N* = 430) and homogenous sample of BC survivors. Due to General Data Protection Regulation (GDRP), it was not possible to obtain patient and treatment data from the cancer registry or electronic health files. The data were provided as self-reported information and therefore physicians reported data are missing. Due to the GDPR, it was also not possible to evaluate the difference between those who did or did not respond to the survey (i.e., responders vs. non-responders’ analysis). This is a cross-sectional study, and no information is available on how unmet needs change over time. Longitudinal studies are needed to assess how unmet needs influence the identified factors. Unmet needs were assessed in the Slovenian sample of cancer survivors during the time of the pandemic, which reflects needs that are unique for the country and the corresponding healthcare system (e.g., accessible hospital parking), and the sensitive time when the study was performed.

### Clinical implications and conclusion

This study has indicated that many breast cancer survivors experience high unmet needs. This can also be attributed to the COVID-19 pandemic when cancer care was disrupted and the fear of the unknown increased. The clinical implications of these findings concern all hospital care teams, including the hospital organization itself. This study highlights the need for more accessible parking and interventions targeting the fear of cancer recurrence, which psychologists in cancer care should incorporate into their practice. The role of family physicians in this stage has not been well defined yet, and at this moment, no clear guidelines exist on how to approach this population. However, family physicians follow these patients through life, are their closest confidants, and have a lot to offer due to a large set of information about their patient’s health history, family situation, and holistic approach to healthcare. Based on our results, we suggest family physicians be aware of cancer survivors who are younger and have more comorbidities since those characteristics can be detected without additional time and were found to be significantly related to high levels of unmet needs. If physicians correctly recognize patients’ unmet needs, communication with them will be more effective and efficient, improving cancer care and treatment-related decisions.

Our results suggest that the high prevalence in our sample identifies the need for informing health service planning and optimizing a model of care for cancer survivors. This includes identification of those at high risk for unmet needs, providing accessible parking spots, the inclusion of ongoing case managers, providing FCR interventions, and improving the current educational curriculum by including cancer survivorship in the training of physicians to better respond to the increasing number of cancer survivors in primary care.

## Data availability statement

The raw data supporting the conclusions of this article will be made available by the authors, without undue reservation.

## Ethics statement

The studies involving human participants were reviewed and approved by National Medical Ethics Committee (no. 0120-25/2019/6) and the Ethics Committee of the Institute of Oncology Ljubljana (EK-OI-16092021). The patients/participants provided their written informed consent to participate in this study.

## Author contributions

All authors contributed to the study conception and design. Material preparation, data collection, and analysis were performed by SM, JP, SB, NB, and ZK-K. The first draft of the manuscript was written by SM and all authors commented on previous versions of the manuscript. All authors contributed to the article and approved the submitted version.

## Funding

The project was funded by the Slovenian Research Agency ARRS (Programs MR-39262 and P3-0339).

## Conflict of interest

The authors declare that the research was conducted in the absence of any commercial or financial relationships that could be construed as a potential conflict of interest.

## Publisher’s note

All claims expressed in this article are solely those of the authors and do not necessarily represent those of their affiliated organizations, or those of the publisher, the editors and the reviewers. Any product that may be evaluated in this article, or claim that may be made by its manufacturer, is not guaranteed or endorsed by the publisher.
