# Supplementary material for: Factors associated with a high level of unmet needs and their prevalence in the breast cancer survivors 1–5 years after post local treatment and (neo)adjuvant chemotherapy during the COVID-19: A cross-sectional study
Source: Front Psychol. 2022 Oct 3;13:969918. doi: 10.3389/fpsyg.2022.969918 (PMC9574393; doi:10.3389/fpsyg.2022.969918)
Supplement: Supplementary file 1 [file Table_1.docx]

Supplement 1.

*Age-adjusted univariate analysis between total number of unmet needs and unmet needs in specific domains with all potential predictors*

| **Variable** | **Total needs** | | **Comprehensive care** | | **Psychological and emotional support** | |
| --- | --- | --- | --- | --- | --- | --- |
|  | OR (95% Cl) | P-value | OR (95% Cl) | P-value | OR (95% Cl) | P-value |
| **Sociodemographic** |  |  |  |  |  |  |
| Marital status (ref. Married)  Partnered^a^  Single, divorced^a^  Widowed^a^ | 1.12 (0.59-2.13)  2.25 (1.11-4.55)  2.02 (0.93-4.38) | 0.73  0.02  0.08 | 1.09 (0.61-1.95)  1.22 (0.67-2.22)  0.87 (0.37-2.03) | 0.78  0.51  0.74 | 1.08 (0.59-1.96)  1.41 (0.76-2.61)  0.92 (0.34-2.45) | 0.81  0.27  0.87 |
| Education (ref. Primary)  Secondary^a^  University, PhD^a^ | 0.37 (0.14-0.93)  0.37 (0.14-0.97) | 0.04  0.04 | 1.26 (0.50-3.19)  1.74 (0.68-4.50) | 0.62  0.25 | 0.37 (0.16-0.91)  0.55 (0.22-1.35) | 0.03  0.19 |
| Employment status (ref. Full-time)  Half-time^a^  Unemployed^a^  Disabled retired^a^  Retired^a^ | 1.90 (1.01-3.56)  1.21 (0.31-4.72)  3.46 (0.72-16.56)  0.99 (0.47-2.12) | 0.05  0.78  0.12  0.99 | 1.03 (0.61-1.75)  0.63 (0.20-2.03)  4.46 (1.16-17.19)  0.66 (0.32-1.37) | 0.90  0.44  0.03  0.26 | 1.19 (0.69-2.05)  0.23 (0.06-0.97)  4.25 (1.26-14.34)  1.30 (0.59-2.88) | 0.53  0.05  0.02  0.51 |
| Place of residence (ref. Urban)  Sub-urban^a^  Rural^a^ | 1.10 (0.69-1.79)  1.241 (0.83-2.40) | 0.67  0.21 | 0.99 (0.62-1.57)  0.89 (0.54-1.48) | 0.97  0.66 | 1.40 (0.87-2.28)  1.72 (1.01-2.92) | 0.18  0.05 |
| Smoking status (ref. Never smoked)  Currently smoking^a^  Past smoker^a^ | 0.81 (0.41-1.60)  1.43 (0.81-2.54) | 0.54  0.22 | 0.50 (0.24-1.01)  1.10 (0.65-1.84) | 0.07  0.74 | 1.34 (0.66-2.73)  1.03 (0.60-1.79) | 0.81  0.91 |
| **Disease and treatment-related** |  |  |  |  |  |  |
| Time since treatment | 0.99 (0.98-1.00) | 0.10 | 0.98 (0.97-1.00) | 0.04 | 0.99 (0.98-1.00) | 0.02 |
| Cancer stage (ref. 0-I)  II^a^  III^a^ | 1.29 (0.77-2.15)  1.64 (0.85-3.16) | 0.33  0.14 | 0.82 (0.50-1.34)  1.33 (0.72-2.46) | 0.43  0.36 | 0.94 (0.55-1.61)  1.20 (0.63-2.25) | 0.83  0.58 |
| Treatment type (ref. None)  Chemotherapy (C)^a^  Radiotherapy (R)^a^  C + R^a^ | 1.35 (0.55-3.31)  0.84 (0.43-1.66)  1.26 (0.63-2.56) | 0.51  0.62  0.51 | 1.88 (0.82-4.32)  1.08 (0.54-2.13)  1.55 (0.79-3.03) | 0.14  0.84  0.20 | 2.55 (1.076.11)  1.14 (0.54-2.42)  1.73 (0.84-3.55) | 0.04  0.73  0.14 |
| Hormonal therapy (yes,no) | 1.22 (0.80-1.87) | 0.36 | 1.57 (1.03-2.39) | 0.04 | 1.08 (0.70-1.67) | 0.73 |
| SCQ-19, comorbidities (ref. ≥ 3)  None^a^  1-2^a^ | 0.21 (0.10-0.45)  0.42 (0.21-0.84) | <0.001  0.01 | 0.21 (0.10-0.42)  0.49 (0.26-0.91) | <0.001  0.03 | 0.31 (0.15-0.64)  0.57 (0.30-1.10) | 0.001  0.10 |
| **Psychosocial** |  |  |  |  |  |  |
| EQ-5D - index, quality of life | 0.01 (0.00-0.04) | <0.001 | 0.02 (0.01-0.08) | <0.001 | 0.01 (0.00-0.03) | <0.01 |
| HADS, anxiety | 1.25 (1.16-1.34) | <0.001 | 1.17 (1.10-1.24) | <0.001 | 1.41 (1.30-1.52) | <0.001 |
| HADS, depression | 1.26 (1.17-1.34) | <0.001 | 1.18 (1.12-1.25) | <0.001 | 1.32 (1.24-1.42) | <0.001 |
| FCRI, fear of cancer recurrence | 1.10 (1.05-1.12) | <0.001 | 1.07 (1.04-1.11) | <0.001 | 1.14 (1.10-1.18) | <0.001 |
| RS-14, resilience | 0.93 (0.91-.095) | <0.001 | 0.95 (0.94-0.97) | <0.001 | 0.93 (0.91-0.95) | <0.001 |
| MPSS-total, social support | 0.96 (0.94-0.98) | <0.001 | 0.98 (0.96-0.99) | <0.001 | 0.95 (0.94-0.97) | <0.001 |

*Notes.* OR, odds ratio. Cl, confidence interval.

^a^Predictor is a dummy variable.
